# Correlates of quality of life in South Korean patients undergoing hematopoietic stem cell transplantation based on the symptom management model

**DOI:** 10.1038/s41598-022-21994-8

**Published:** 2022-11-16

**Authors:** EunJin Jo, Sanghee Kim, Hyangkyu Lee, Seok Lee

**Affiliations:** 1 Department of Nursing, College of Nursing, Yonsei University, 50-1 Yonsei-ro, Seodaemun-gu, Seoul, 03722 South Korea; 2 Department of Nursing, College of Nursing, Mo-Im Kim Nursing Research Institute, Yonsei University, 50-1 Yonsei-ro, Seodaemun-gu, Seoul, 03722 South Korea; 3 Department of Hematology, Seoul St. Mary’s Hospital, Catholic University of Korea, 222 Banpo-daero, Seocho-gu, Seoul, 06591 South Korea

**Keywords:** Health care, Oncology, Signs and symptoms

## Abstract

While hematopoietic stem cell transplantation (HSCT) has led to higher survival rates, the number of patients experiencing adverse reactions is also increasing. Based on the symptom management model, we aimed to analyze the relationships between symptom experience, symptom management strategies, self-management behavior, and quality of life among patients undergoing HSCT in South Korea and to identify the factors affecting their quality of life. The data of 67 conveniently sampled patients undergoing HSCT at a university-affiliated hospital, for the period from March 23 to June 7, 2016, were collected using a self-reported structured questionnaire. Data were analyzed using Pearson’s correlations and multiple regression analysis. Quality of life showed a significant negative correlation with symptom experience and significant positive correlations with symptom management strategies. Factors influencing quality of life included symptom distress, symptom management strategies, and self-management behavior; these explained 39.4% of the variance. To improve quality of life in patients undergoing HSCT, the efficient management of multiple co-existing symptoms is important. There is a need for tailored nursing interventions based on a patient-focused and person-centered approach for patients undergoing HSCT.

## Introduction

In hematopoietic stem cell transplantation (HSCT), normal hematopoietic stem cells are injected to re-establish hematopoietic capability, which helps treat conditions such as bone marrow failure, malignant bone marrow-origin diseases, and bone marrow abnormalities. HSCT, thus, makes the treatment of hemopathies, solid cancers, and refractory genetic diseases possible^1^. More than 1.5 million HSCT procedures have been performed at over 1500 transplant centers worldwide^2^. In South Korea, as of 2021, 36,592 patients had been reported to have undergone HSCT; this treatment is on the rise for reasons such as advancements in transplant technology and the expansion of medical insurance coverage^3^. As the number of such transplantations performed has increased, survival rates have improved. However, the number of patients experiencing adverse reactions to HSCT is also increasing^4^.

Pre-transplantation procedures include chemotherapy and radiotherapy to suppress immune and hematopoietic functions and destroy cancer cells. After transplantation, patients are hospitalized in a protective isolation unit for the engraftment of hematopoietic stem cells^5^. The average duration of hospitalization in a protective isolation unit in South Korea is about one month; this is the most critical period for patients^6^. In the first two weeks of hospitalization, patients are in the physically debilitating state of extreme immunosuppression, during which they may contract various infections. They may also experience physical discomfort in the form of sore throat, pain caused by mouth ulcers, nausea and vomiting, salivary hypofunction or hyperfunction, diarrhea, fatigue, shortness of breath, coughing, headache, tingling of hands and feet, and skin pigmentation, as well as emotional symptoms such as frustration, depression, and helplessness^7^. Thus, they may experience a wide range of adverse events associated with HSCT, from minimal side effects to life-threatening issues. Based on the manageability of their symptoms, patients face physical and mental risks that threaten their quality of life^8^.

Previous studies on the correlation between adverse symptoms and quality of life in patients undergoing HSCT found that symptoms had a significant effect on quality of life and that effective management positively influenced this^9^. As effective symptom management can also reduce pain and improve quality of life, the International Association of Clinical Research Nurses noted that symptom management holds priority in their agenda. Studying symptom management among patients who are undergoing HSCT involves a medical approach to specific symptoms related to fatigue, stomatitis, and neutropenia^10^. While there are studies on symptom management in patients with multiple myeloma and acute myeloid leukemia^11,12^, there is a lack of research on the management of concurrent symptoms in patients undergoing HSCT. Therefore, it is necessary to understand the symptom experience of patients undergoing HSCT and to provide effective symptom management to improve their quality of life. Thus, our study is based on the application of nursing theory.

Among the various symptom-related theories, we chose the symptom management model, a middle-range theory developed by Dodd^13^, as the conceptual framework for this study. This theory encompasses symptom experience, symptom management strategies, and symptom outcomes considered the result of symptom management strategies and symptom experience. In symptom management, it is important to measure the frequency, intensity, and pain degree for each symptom experience. Symptom management strategies involve trial and error, with positive consequences such as symptom relief, reduced pain, prevention of recurrence, and improvement in quality of life and negative consequences such as symptom recurrence, consistent or increased pain, and reduced quality of life. Thus, the emphasis is on adherence to improve the effectiveness of symptom management strategies. Although there is a risk of nonadherence to treatment in the presence of multiple symptom management strategies, adherence remains important for symptom management and outcomes. Self-management among patients indicates a proper beginning to a symptom management strategy and reveals more functional, effective, and creative strategies to improve quality of life.

Previous studies based on the symptom management model have focused on children with tumors^14,15^, patients with solid cancers^16^, patients with uterine cancer^17^, and family caregivers of people with cancer^18^. The inconsistent symptom experiences reported in these studies can be attributed to the diverse disease types studied; as the symptom experiences became more severe, the effectiveness of symptom management strategies decreased and quality of life reduced ^14–18^. Despite this issue, studies have shown that the symptom management model is useful in that it enhances the subjective experience of patients’ symptoms and provides a framework for recognizing and analyzing signs that are abnormal indicators of diseases that can be found by individual patients or others. However, there have been no studies applying this theory to patients undergoing HSCT.

**Figure 1:**
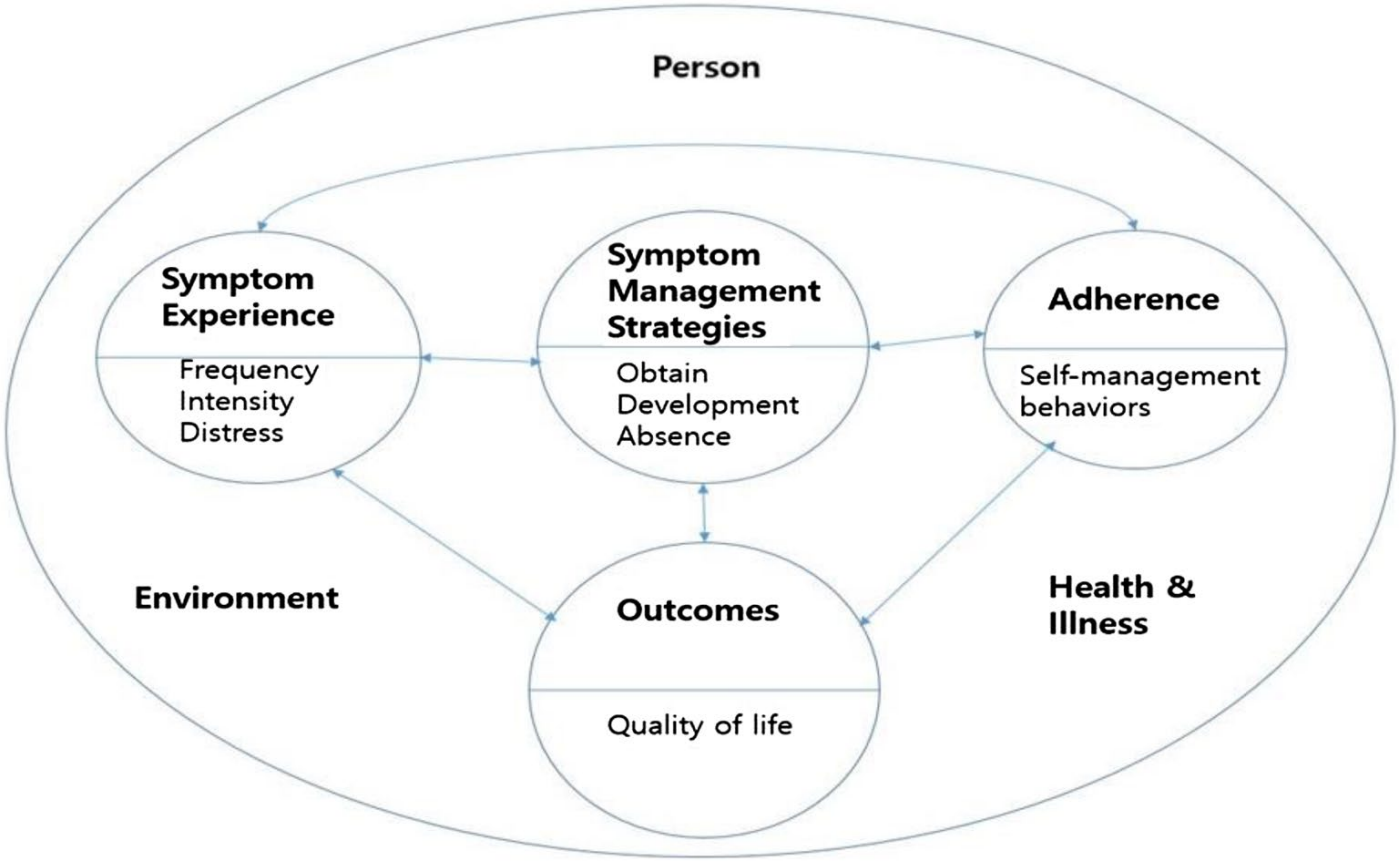
Conceptual Framework.

Against this background, in this study, we aim to provide basic data to develop a nursing intervention for effective symptom management for patients undergoing HSCT by identifying the factors associated with symptom experience, symptom management strategies, self-management behavior, and quality of life, based on the symptom management model^13^ within an integrated theoretical framework. Figure 1 shows this framework, identifying improvements in the quality of life of patients undergoing HSCT.

## Methods

### Design

This study employed a cross-sectional, descriptive, correlational design. We examined the relationships between symptom experience, symptom management strategies, self-management behavior, and quality of life among patients undergoing HSCT, analyzed their effects, and identified other factors affecting quality of life.

We partially revised the symptom management model to ensure the economic feasibility of the research model and analysis and identify items helpful to nursing practice. Symptom experience, symptom management strategies, and self-management behavior were all included to assess individual aspects, including patients’ demographic characteristics; environmental aspects indicating the degree of support family or medical staff offered with regard to self-management and health; and disease aspects associated with HSCT; as well as factors influencing the symptom outcomes. Symptom experience, symptom management strategies, and self-management behavior are related, and this study focuses on quality of life as a symptom outcome. Therefore, factors such as cost, morbidity, prevalence, and mortality as symptom outcomes were excluded. Thus, our theoretical framework comprises subject aspects, environmental aspects, health and disease aspects, symptom experience, symptom management strategies, self-management behavior, and quality of life.

### Participants

The participants were conveniently sampled adult patients undergoing HSCT at S Hospital of C University in Seoul, South Korea. We included data for the period from March 23 to June 7, 2016. The inclusion criteria were as follows: (1) aged 20 years or older; (2) hospitalized in a protective isolation unit; (3) having undergone HSCT 1–14 days previously; and the absence of brain, heart, or kidney diseases, which could affect patients’ symptoms. The required number of participants for multiple linear regression was calculated using G*Power version 3.1, with six variables based on the expected large effect size (0.35), significance level (0.05), and power (0.9); the minimum sample size was calculated to be 57 patients, and considering a dropout rate 20%, we aimed to recruit 68 patients. Among the 68 retrieved questionnaires, one was excluded because of missing responses; the remaining 67 were used in the final analysis.

## Variables

### Patient characteristics

Medical records and self-report questionnaires were used to investigate patients’ general, environmental, and disease characteristics. General characteristics included gender, age, education, marital status, job, and religion. Environmental characteristics included perceived self-management ability and support for self-management from medical staff and families. The environmental characteristic of the degree of recognition of their support system was self-reported; it was calculated from the subjective response of each patient and recorded on a 10-point scale. Disease characteristics included diagnosis, type, and source of HSCT.

### Symptom experience

We used the Symptom Frequency, Intensity and Distress questionnaire for stem cell transplantation^19^. This instrument comprises 25 items. A four-point scale (1: “not severe at all” to 4: “very severe”) was used to report the intensity and distress of each symptom. The higher the score, the greater the severity of the symptom experience. Regarding reliability, the Cronbach’s alpha at the time of development and in the present study was 0.81 and 0.93, respectively.

### Symptom management strategies

We used the Appraisal of Self-Care Agency Scale-Revised^20^, modified to measure symptom management strategies and supplemented by five expert validations. The instrument comprised 15 items and three subsections—acquisition of symptom management strategy, symptom management strategy development, and absence of symptom management strategy. The items were rated on a five-point scale from 1 (not at all) to 5 (very much), with higher scores suggesting greater symptom management strategies. Regarding reliability, Cronbach’s alpha at the time of development and in the present study was 0.90 and 0.76, respectively.

### Self-management behavior

We used the framework for self-management behavior in patients undergoing HSCT revised by Kim^21^. General self-management comprises 18 items and five subsections. Symptom management comprises 35 items and 13 subsections.and The items were rated on a four-point scale from 1 (not implemented at all) to 4 (always well implemented). Higher scores indicated better implementation of self-management. The Cronbach’s alpha at the time of development was 0.65–0.92 for general self-management and 0.64–0.88 for symptom management. In this study, the Cronbach’s alpha was 0.90 for the total tool.

### Quality of life

We used the South Korean version of the Functional Assessment of Cancer Therapy-Bone Marrow Transplantation (Version 4)^22^. This instrument comprises 50 items and five subsections. The items were rated on a four-point scale from 1 (not at all) to 4 (quite a lot). Higher scores indicated better quality of life. The Cronbach’s alpha at the time of development and in the present study was 0.94 and 0.82, respectively.

### Ethical considerations

This study was approved by the Research Ethics Council of C University Hospital in Seoul (No. KC16QASI0243). Prior to data collection, the researcher gave the patients an explanation that covered the purpose of the study, anonymity and confidentiality, and that they could stop answering the questionnaire at any point. The patients were also informed that their electronic medical records would be used only for the collection of items related to disease-related characteristics. Subsequently, the patients provided written informed consent. Data from the electronic medical records were managed with unique identification numbers to avoid patient identification.

### Data collection

We requested the cooperation of the nursing department of the hospital to collect data. Further, the researcher explained the purpose of the study to the head nurse of the HSCT ward and the faculty of hematology and requested their cooperation. Subsequently, we visited patients who were admitted to the HSCT ward and met the inclusion criteria. Patients responded to the survey only once within 1–14 days of HSCT. The survey took about 15 min to complete, and we recorded the disease-related characteristics from the electronic medical records. All methods were performed in accordance with the relevant Strengthening the Reporting of Observational Studies in Epidemiology guidelines and regulations. All methods were performed in accordance with the relevant guidelines and regulations by including a statement in the methods.

### Data analysis

Participants’ general, environmental, and disease-related characteristics; symptom experience; symptom management strategies; self-management behavior; and quality of life were analyzed using frequency, percentage, mean, and standard deviation.

Symptom experience, symptom management strategies, self-management behavior, and quality of life according to the participants’ general, environmental, and disease-related characteristics were analyzed using the t-test and one-way analysis of variance.

The correlations between symptom experience, symptom management strategies, self-management behavior, and quality of life were analyzed using correlation analysis.

Multiple regression analysis was used to identify factors affecting quality of life.

SPSS 21.0 (IBM SPSS Statistics for Windows, Version 21.0. IBM Corp., Armonk, NY, USA) was used for all analyses.

## Results

### General, environmental, and disease-related characteristics

The participants’ average age was 44.58 years. Of the sample, 67.2% were men and 32.8% were women. We determined that 91.1% had an educational level higher than high school and 68.7% were married. Furthermore, 41.8% were office workers and 43.3% had no religion. Participants’ subjective perception of their self-management ability scored an average of 6.49 points out of 10. As for the family members participants thought would offer support with self-management, 49.3% mentioned spouses and 23.9% mentioned parents. The subjective perception of receiving self-management support from the family scored an average of 7.54 points out of 10. Regarding the medical team members participants thought would offer support with self-management, 53.7% mentioned nurses and 41.8% mentioned doctors. The subjective perception of receiving support from medical staff for self-management scored 8.49 points out of 10. As for the diagnosis, 46.3% of the participants had leukemia and 17.9% had myelodysplastic syndrome. Regarding type, sibling transplantation accounted for 29.9% and unrelated and autologous for 26.9% (Table 1).

**Table 1:** In variable of Symptom Management Model according to Characteristic.

Characteristics	Variable	Category	N	%	Mean±SD	Symptom Frequency	Symptom Intensity	Symptom Distress	Symptom management Strategies	Self-management Behavior	Quality of Life
						t or F (*p*)	t or F *(p*)	t or F (*p*)	t or F (*p*)	t or F (*p*)	t or F (*p*)
Demo graphical	Gender	Male	45	67.2		−1.044 (0.262)	−0.999 (0.322)	−1.414 (0.162)	−0.770 (0.444)	−1.683 (.097)	−0.532 (.597)
		Female	22	32.8							
	Age(years)				44.58 ± 13.48	0.302 (0.876)	0.461 (0.764)	0.061 (0.656)	1.601 (0.185)	0.659 (0.623)	0.806 (0.526)
	Education	≤Middle school^a^	6	9		0.839 (0.477)	2.600 (0.060)	1.677 (0.181)	1.939 (0.132)	3.979 (0.012)^*^ b<d^**^	0.894 (0.449)
		High school^b^	19	28.4							
		University student^c^	10	14.9							
		≥University school^d^	32	47.8							
	Marital status	Married	46	68.7		2.080 (.133)	1.867 (.163)	1.654 (.199)	0.424 (.132)	2.786 (.069)	0.990 (.377)
		Single	21	31.3							
	Job	Employed	28	41.8		1.199 (0.317)	1.430 (.242)	1.696 (0.411)	0.953 (.421)	0.511 (.676)	1.866 (.144)
		Unemployed	25	37.3							
		Self employed	8	11.9							
		Student	6	9							
	Religion	None	29	43.3		0.447 (0.720)	0.698 (0.557)	0.973 (0.411)	0.067 (0.977)	2.524 (0.066)	0.746 (0.529)
		Christianity	17	25.4							
		Catholic	12	17.9							
		Buddhist	9	13.4							
Environmental	Perceived Self management ability				6.49 ± 1.99	0.156 (0.207)	0.076 (0.540)	0.085 (0.492)	0.264 (0.031)	0.230 (0.061)	0.058 (0.639)
	Support for Self management (Family)	Spouse Parents Brother/Sister Son/Daughter Others	33 16 7 7 4	49.3 23.9 10.4 10.4 6.	7.54 ± 2.02	0.837 (0.507)	.0802 (0.528)	0.880 (.481)	0.455 (.769)	0.462 (0.763)	0.602 (0.662)
	Support for Self management (Medical)	Nurse^a^ Doctor^b^ Other^c^		36 53.7 2 41.8 3 4.5	8.49 ± 1.33	0.966 (0.386)*	1.228 (0.300)	0.829 (0.441)	3.570 (0.034)* ^a,b>c**^	2.986 (0.058)	1.279 (0.285)
Clinical	Diagnosis	Leukemia	31	46.3		1.881 (0.089)	2.355 (0.130)	2.131 (0.054)	1.747 (0.115)	0.847 (0.553)	0.554 (0.790)
		MDS^*^	12	17.9							
		MM^†^	9	16.4							
		Lymphoma	8	11.9							
		SAA^‡^	4	7.5							
	Type of HSCT^§^	Sibling	20	29.9		0.085 (0.968)	0.538 (0.658)	0.631 (0.598)	0.532 (0.662)	2.66 (0.101)	0.392 (0.759)
		Unrelated	18	26.9							
		Autologous	18	26.9							
		Family Mismatched	11	16.4							
	Stem Cell Source of HSCT^§^	PBSC^¶^	59	88.1		0.405 (0.750)	0.263 (0.852)	0.363 (0.780)	1.161 (0.332)	0.291 (0.831)	0.551 (0.649)
		BM^#^	5	7.5							
		Cord	2	3							
		PBSC^¶^+BM^#^	1	1.5							

Post hoc : *p < .05, **p < .001.

### Symptom experience, symptom management strategies, self-management behavior, and quality of life

The symptom experience of patients undergoing HSCT consisted of frequency, intensity, and distress. The frequency of symptoms of patients undergoing hematopoietic stem cell transplantation was the highest among 25 items, with anorexia 64 patients (95.5%) and taste change 64 patients (95.5%) as the most frequent 5 items, followed by sleep disturbance. (80.6%), dry mouth (80.6%), fatigue (79.1%), etc. In the symptom intensity of patients undergoing hematopoietic stem cell transplantation, anorexia was the highest with an average score of 3.03 points, followed by changes in taste 3.01 points, sleep disturbance 2.57 points, dry mouth 2.45 ± 1.06 points, and fatigue 2.42 point. Anorexia was the highest with an average score of 2.91 points, followed by changes in taste 2.91 points, sleep disturbances 2.69 points, and dry mouth 2.48 points. In the symptom distress in patients undergoing hematopoietic stem cell transplantation, followed by vomiting 2.45 points. In the symptom experience of patients undergoing hematopoietic stem cell transplantation, anorexia had the highest average score in all three items of frequency, intensity, and distress. The mean symptom management strategy score was 3.55 points (range 1–4). By sub-items of the symptom management strategy, obtaining the symptom management strategy scored the highest with an average score of 3.81 points, followed by the development of the symptom management strategy with an average score of 3.68 points, and the absence of a symptom management strategy with an average score of 3.00 points. The average score for self-management behavior was 2.74 points (range 1–4). The average score for quality of life was 1.95 points (range 1–4) (Table 2).

**Table 2:** Descriptive Statistics of Symptom Experience, Symptom Management Strategies, Self-management Behavior, Quality of Life **(N = 67)**.

Variable	Categories			
Symptom experience	Symptom	Frequency N(%)	Intensity M ± SD	Distress M ± SD
	Loss of appetite	64 (95.5)	3.03 ± 0.79	2.91 ± 0.81
	Changes of taste	64 (95.5)	3.01 ± 0.84	2.91 ± 0.88
	Sleeping disturbance	54 (80.6)	2.57 ± 1.00	2.69 ± 1.05
	Mouth dryness	54 (80.6)	2.45 ± 1.06	2.48 ± 1.04
	Fatigue	53 (79.1)	2.42 ± 0.94	2.31 ± 0.97
	Nausea	49 (73.1)	2.40 ± 1.10	2.43 ± 1.14
	Vomiting	49 (73.1)	2.33 ± 1.04	2.45 ± 1.17
	Diarrhea	48 (71.6)	2.28 ± 1.08	2.40 ± 1.12
	Anxiety	48 (71.6)	2.21 ± 1.02	2.21 ± 1.04
	Depression	46 (68.7)	2.21 ± 1.05	2.18 ± 1.06
	Difficulty to concentrate	45 (67.2)	2.04 ± 0.93	1.93 ± 0.91
	Pain	44 (65.7)	2.10 ± 1.03	2.18 ± 1.09
	Reduced mobility	43 (68.7)	2.16 ± 1.10	2.16 ± 1.12
	Changed Body image	39 (58.2)	1.79 ± 0.91	1.76 ± 0.91
	Mouth sores	38 (56.7)	2.15 ± 1.12	2.30 ± 1.24
	Loss of hair	38 (56.7)	2.10 ± 1.14	1.79 ± 0.99
	Skin changes	38 (56.7)	1.67 ± 0.81	1.61 ± 0.76
	Difficulty in remembering	37 (54.7)	1.88 ± 0.91	1.85 ± 0.93
	Changing eye	35 (52.2)	1.73 ± 0.88	1.72 ± 0.88
	Chilling	33 (49.3)	1.66 ± 0.90	1.70 ± 0.97
	Constipation	32 (47.8)	1.73 ± 0.99	1.76 ± 1.05
	Fever	29 (43.3)	1.63 ± 0.90	1.69 ± 0.96
	Sexuality discomfort	16 (23.9))	1.38 ± 0.79	1.38 ± 0.81
	Coughing	13 (19.4)	1.22 ± 0.60	1.19 ± 0.61
	Difficulty in breathing	13 (19.4)	1.18 ± 0.49	1.25 ± 0.64

Variables	Categories	Range	M ± SD	
Symptom management strategiee	Symptom management strategies	1 ~ 4	3.81 ± 0.59	
	Symptom management strategies development	1 ~ 4	3.68 ± 0.57	
	Symptom management strategies absence	1 ~ 4	3.00 ± 0.61	
	Total	1 ~ 4	3.55 ± 0.45	
Self-management behavior	General self-management	1 ~ 4	3.09 ± 0.39	
	Symptom management	1 ~ 4	2.59 ± 0.90	
	Total	1 ~ 4	2.74 ± 0.89	
Quality of life	Physical	1 ~ 4	1.98 ± 0.87	
	Social	1 ~ 4	2.24 ± 0.63	
	Emotional	1 ~ 4	2.75 ± 0.77	
	Functional	1 ~ 4	2.07 ± 0.88	
	BMT subscale	1 ~ 4	1.59 ± 0.34	
	Total	1 ~ 4	1.95 ± 0.32	

### Differences according to patient characteristics

There were statistically significant differences in self-management behavior according to the degree of education (F = 3.979, *p* < 0.05). Self-management performance was higher among college graduates compared to high school graduates. For symptom experience, symptom management strategies, and self-management behavior according to the patients’ environmental characteristics, there were statistically significant differences. There was a statistically significant difference in symptom management strategies according to the medical staff participants thought would assist them with symptom management (F = 3.570, *p* < 0.034). Higher symptom management strategies were found with nurses and doctors than with other medical staff. However, quality of life was not statistically significantly different according to any of the variables (Table 1).

### Relationships between symptom experience, symptom management strategies, self-management behavior, and quality of life

The correlations between symptom experience, symptom management strategies, self-management behavior, and quality of life were as follows. The frequency and intensity of symptom experience (r = 0.898, *p* < 0.001), frequency and pain of symptom experience (r = 0.879, *p* < 0.001), and intensity and pain of symptom experience (r = 0.977, *p* < 0.001) showed statistically significant positive correlations, suggesting that the frequency, intensity, and pain of symptom experience increased together. Frequency of symptom experience and quality of life (r = −0.378, *p* < 0.001), intensity and quality of life (r = −0.450, *p* < 0.001), and pain and quality of life (r = −0.469, *p* < 0.001) showed statistically significant negative correlations, suggesting that the lower the symptom experience, the higher the quality of life. A patient’s symptom management strategy and quality of life (r = 0.323, *p* < 0.001) and self-management behavior and quality of life (r = 0.362, *p* < 0.001) showed statistically significant positive correlations, suggesting that the better the symptom management strategy and the better the self-management, the higher the quality of life (Table 3).

**Table 3:** Correlations between Symptom Experience, Symptom Management Strategies, Self-Management Behavior, Quality of Life **(N = 67)**.

Variables		Quality of life	Symptom experience			Symptom management strategies		Self-management behavior
			Frequency	Intensity	Distress			
Quality of life		1						
Symptom experience	Frequency	− 0.378 (0.002)**	1					
	Intensity	− 0.450 (< .001)**	0.898 (< 0.001)**	1				
	Distress	− 0.469 (< .001)**	0.879 (< 0.001)**	0.977 (< 0.001)**	1			
Symptom management strategies		0.323 (0.008)**	0.042	− 0.091	− 0.045	1		
Self-management behavior		0.362 (0.003)**	0.117	0.087	0.068	0.232		1

**p < .01.

### Factors influencing quality of life

We conducted multiple regression analysis to identify the factors affecting participants’ quality of life. The regression model was significant (F = 15.307, *p* < 0.001), and the tolerance limits and variance inflation factor values were identified to test for multicollinearity. The variance inflation factor of the main independent variable was ≤ 1.1, indicating no multicollinearity. Symptom experience (frequency, intensity, and distress), symptom management strategies, and self-management behavior influenced quality of life, with an explanatory power of 39.2%. The greater the symptom management strategy (β = 0.343, *p* < 0.001), the better the self-management behavior (β = 0.221, *p* = 0.029). Further, the lower the pain in symptom experience (β = −0.482, *p* < 0.001), the greater its influence on quality of life (Table 4).

**Table 4:** The influencing factors of quality of life based on Symptom Management Model **(N = 67)**.

Factors	B	SE	β	T	*P*
Constants	24.201	17.675			
Symptom experience (distress)	− .522	.104	− .482	− 5.014	.000**
Self-management behavior	.344	.099	.343	3.469	.001**
Symptom management strategies	.547	.244	.221	2.239	.029*

F = 15.307**, R^2^ =0.442, Cum R^2^ =0.394

*p < .05, **p < .001.

## Discussion

Based on the symptom management model, we identified correlations between symptom experience, symptom management strategies, self-management behavior, and quality of life. Further, we determined that the factors influencing patients’ quality of life were symptom experience, symptom management strategies, and self-management behavior.

It was observed that the higher the frequency, intensity, and pain of symptoms, the lower patients’ quality of life with respect to symptom experience. These findings are consistent with those of a previous study on the changes in quality of life up to one year after transplantation^9^. In that study, it was reported that the higher the scores for fatigue, anorexia, and pain, the lower the quality of life. Furthermore, as reported in studies examining the symptoms and quality of life of patients undergoing HSCT^4,23^, regular evaluation and management of symptoms are important as the degree of symptom experience directly affects quality of life. As intervention in symptom experience can improve quality of life, appropriate management is needed. Thus, we recommend active nursing interventions to improve the quality of life of patients undergoing HSCT.

Moreover, we observed that the higher the symptom management strategies regarding HSCT, the better the quality of life. These results are consistent with Chou’s findings pertaining to patients with cancer based on the symptom management model, which reported moderate quality of life for groups using an average of two or three self-management strategies^16^. Although symptom management strategies and symptom outcomes were measured using a different tool in this study, the quality of life is expected to improve as patients’ functional and emotional states change positively through proper symptom management. Therefore, studies regarding the development of personalized nursing strategies, interventions, and clinical application should be conducted to improve quality of life, a symptomatic outcome of HSCT.

We also determined that the better the self-management behavior, the higher the quality of life. A study on patients with cervical cancer reported that the greater the symptom experience, the lower the self-management efficiency^17^. However, there was a U-shaped increase in efficiency as the symptom experience decreased over time. Here, we examined the data of a different group of patients and found no significant difference between symptom experience and self-management behavior. Thus, it is necessary to examine the extent of symptom experience and self-management behavior and their correlation with quality of life in patients who are undergoing HSCT through repeated further studies.

Regarding the factors influencing quality of life, the lower the symptom distress and the better the symptom management strategies, the better the self-management behavior, supporting some findings of the symptom management model. These include the conclusion that symptom management strategies result in relieving symptoms, reducing symptom distress, preventing recurrence, and improving quality of life. For about a month on their own without a caregiver in a protective isolation unit, patients may have a different level of need; therefore, a different approach to implementing symptom management strategies and self-management to cope with various symptoms is required^24^. Thus, patient-centered care and emotionally supportive care in isolation units, such as visit times and outside food intake, are instrumental in improving the quality of life of patients undergoing HSCT. In addition, nurses and doctors were the supporting medical faculty for symptom management of patients undergoing HSCT. These findings are consistent with those of Kang^6^, who found that the most contacted medical staff during HSCT in the isolation unit were nurses, who spent the maximum time with patients. Thus, there is a need for medical staff to actively manage timing, circumstances, and responses to improve patient management strategies^25^. In order to alleviate symptoms in patients undergoing HSCT, yoga^26^, exercise^27^, and music therapy^28^ have been utilized, with many reports of their efficacy. The data derived from this study can be used to expand upon such existing interventions for more effective symptom management. From previous studies interventions^26–28^ have conducted to alleviate overall symptoms meanwhile, by data from this study it can help you find out the symptoms to focus from now on and it could be referred to when it comes to provide symptom management interventions.

Our findings support the theory that symptom outcomes arise from symptom experience, symptom management strategies, and adherence as presented in the symptom management model. This is also consistent with previous theories stating that symptom experience and symptom management strategies are supported by symptom outcomes^16^. We present the correlation of a patient’s symptom experience, symptom management strategies, and self-management behavior with their quality of life. However, the impact analysis of symptom results showed that only some factors were supported for general, environmental, disease, symptom experience, symptom management strategy, and self-management behavior variables. Thus, this study failed to measure various variables that affect the quality of life of patients undergoing HSCT. It is necessary to develop a professional and individualized nursing intervention to establish and implement a symptom management strategy to improve patients’ quality of life based on the various aspects of the symptom management model and the simultaneous occurrence of symptom experiences.

Although the hospital included in this study is responsible for most cases of HSCT in South Korea, this study is limited by its cross-sectional descriptive design, sampling only those patients admitted to the HSCT ward during the study period. Therefore, care must be taken when generalizing or expanding the findings. While the symptom management model—the theoretical framework of this study—describes that all domains affect each other, we only investigated the social environment, not the cultural factors. Nevertheless, the causal relationships between relevant variables were investigated in patients undergoing HSCT based on the symptom management model. This is significant in that it offered a theoretical basis to develop the most effective interventions to improve the quality of life of patients undergoing HSCT.

## Conclusion

We identified correlations between symptom experience, symptom management strategies, self-management behavior, and quality of life and identified that symptom experience (distress), symptom management strategies, and self-management behavior were the factors affecting quality of life. While this study was conducted to identify the influence of factors on patients undergoing HSCT and provide a basis for nursing intervention development, we also found that for factors affecting patients’ quality of life, the greater the number of symptomatic experience, the lower the quality of life. The better the symptom management strategies and the better the self-management behavior, the higher the quality of life. Through this study, we aimed to provide a foundation for nursing intervention development by describing the symptom experience of patients undergoing HSCT, their symptom management strategies, symptom outcomes, and the factors affecting quality of life as the symptom outcome**.** These results suggest that it is necessary to develop individualized interventions to regularly evaluate and manage symptom experience and construct a clinical nursing plan to improve the quality of life of patients undergoing HSCT. Oncology nurses need to conduct continuous patient education and provide supportive care to enable patients to consistently manage themselves.

Based on this study, we recommend the following. First, to improve the quality of life of patients undergoing HSCT, further research is needed to develop a nursing intervention program for symptom management. Second, it is necessary to expand the target population to children and adolescents undergoing HSCT, to determine whether there are differences from adults undergoing this procedure and to develop customized nursing research scales. Third, a longitudinal study is needed to further investigate the causes of self-management of symptoms and improved quality of life in patients as well as survivors after transplantation or patients with graft versus host disease.

## Data Availability

The datasets analyzed in the current study are available from the corresponding author on reasonable request.
